# RGS4 impacts carbohydrate and siderophore metabolism in *Trichoderma reesei*

**DOI:** 10.1186/s12864-023-09467-2

**Published:** 2023-07-03

**Authors:** Miriam Schalamun, Eva Maria Molin, Monika Schmoll

**Affiliations:** 1 AIT Austrian Institute of Technology GmbH, Bioresources Unit, Center for Health & Bioresources, Konrad Lorenz Strasse 24, Tulln, 3430 Austria; 2 Division of Terrestrial Ecosystem Research, Centre of Microbiology and Ecosystem Science, University of Vienna, Djerassiplatz 1, Vienna, 1030 Austria

**Keywords:** *Trichoderma reesei*, *Hypocrea jecorina*, Regulator of G-protein signaling, Cellulase, Nutrient sensing, Light response, Storage carbohydrates, Iron homeostasis, Siderophore

## Abstract

**Background:**

Adaptation to complex, rapidly changing environments is crucial for evolutionary success of fungi. The heterotrimeric G-protein pathway belongs to the most important signaling cascades applied for this task. In *Trichoderma reesei,* enzyme production, growth and secondary metabolism are among the physiological traits influenced by the G-protein pathway in a light dependent manner.

**Results:**

Here, we investigated the function of the SNX/H-type regulator of G-protein signaling (RGS) protein RGS4 of *T. reesei*. We show that RGS4 is involved in regulation of cellulase production, growth, asexual development and oxidative stress response in darkness as well as in osmotic stress response in the presence of sodium chloride, particularly in light. Transcriptome analysis revealed regulation of several ribosomal genes, six genes mutated in RutC30 as well as several genes encoding transcription factors and transporters. Importantly, RGS4 positively regulates the siderophore cluster responsible for fusarinine C biosynthesis in light. The respective deletion mutant shows altered growth on nutrient sources related to siderophore production such as ornithine or proline in a BIOLOG phenotype microarray assay. Additionally, growth on storage carbohydrates as well as several intermediates of the D-galactose and D-arabinose catabolic pathway is decreased, predominantly in light.

**Conclusions:**

We conclude that RGS4 mainly operates in light and targets plant cell wall degradation, siderophore production and storage compound metabolism in *T. reesei*.

**Supplementary Information:**

The online version contains supplementary material available at 10.1186/s12864-023-09467-2.

## Background

Fungi have to adapt to their environment to survive and succeed in competition. Such environmental cues might be the available nutrients, light, defense against competitors or finding a mating partner. Therefore, complex sensing and signaling pathways exist, one of the most important one being heterotrimeric G-protein signaling [1], which profoundly impacts physiological reactions and adaptation to the environment of fungi, from growth and reproduction to secondary metabolism and pathogenicity [2, 3].

The steps of signal transmission from sensing at the plasma membrane to the actual output in terms of enzyme or secondary metabolite production, growth or accumulation of storage compounds and other physiological adaptations are complex and integrate reactions to multiple environmental cues. Thereby, the individual connections from receptors to transmitters to kinases and ultimately transcription factors are only known for very few pathways and mostly only in one model organism. Especially the contributions of RNA- and protein stability, posttranscriptional and posttranslational regulations are often difficult to interpret and integrate into a mechanistic model.

The filamentous ascomycete *Trichoderma reesei* [4, 5] is among the most prolific producers of homologous and heterologous enzymes, especially plant cell wall degrading carbohydrate active enzymes (CAZys), and performance proteins in industry [6, 7]. Recent genome sequencing efforts of the prototypical wild-type QM6a yielded a complete high quality genome [8, 9] and evolutionary analyses revealed an unexpectedly high proportion of CAZyme genes to be acquired to *Trichoderma* through horizontal gene transfer (HGT) [10, 11]. *T. reesei* has become a model organism for plant cell wall degradation in fungi [4, 12], but also for light modulated substrate degradation and enzyme production [13, 14]. The latter phenomenon was investigated in detail in *T. reesei* and connections of light response to the heterotrimeric G-protein pathway, growth, sexual development [15] and secondary metabolism were detected [13, 16, 17]. The light response pathway of *T. reesei* comprises the photoreceptors BLR1 and BLR2, which represent GATA-type transcription factors as well as ENV1, a PAS/LOV domain protein [18, 19]. To achieve its widespread impacts on fungal physiology, diverse signaling pathways are integrated with light response, which involves influences on epigenetic events, posttranscriptional and posttranslational modifications (especially phosphorylation) and protein stability [20, 21].

The function of the G-protein pathway in enzyme biosynthesis was shown to be light dependent [13], which is in agreement with the crucial function of numerous protein kinases, including the cAMP dependent protein kinase A, in light response [22, 23]. The nodes of interaction between the light response pathway and nutrient- and mating partner sensing by the G-protein pathway are still under investigation, although the phosducin-like protein PhLP1 and adenylate cyclase [24] were proposed to play a role in signal integration.

As in most ascomycetes, the G-protein complex in *T. reesei* consists of three alpha-, one beta- and one gamma-subunit [25]. Upon binding of a ligand to a G-protein coupled receptor (GPCRs) the confirmation of the heterotrimeric G-protein complex changes and G-alpha bound GDP is exchanged to GTP [26]. The activated G-proteins dissociate, leading to a free alpha subunit and beta-gamma complex which are now able to transmit downstream signals. The intrinsic GTPase activity of the G-alpha subunits causes GTP hydrolysis to GDP and reassociation of the complex and termination of signal [27, 28].

Regulator of G-protein signaling (RGS) proteins modulate the activity of the heterotrimeric G-protein pathway by accelerating the GTPase activity of the G-alpha subunits [29, 30]. This GTPase activity leads to deactivation of the G-alpha subunit and hence to termination of the transmitted signal [30–32].

RGS proteins are typically regulated at the level of transcription, epigenetic regulation, expression, localization and stability, but not through binding of a ligand. Thereby, phosphorylation by protein kinase A influences localization and stability of RGS proteins. Additionally, feedback mechanisms due to interactions of RGS proteins with their regulating transcription factors are proposed [33]. Besides the impact of RGS proteins on G-alpha subunits, also functions outside this pathway, including activation of MAPkinase signaling are known [34].

In *T. reesei*, the G-protein signaling cascade is well described with respect to its role in enzyme production with characterizations of the G-alpha, -beta and -gamma subunits and a few GPCRs [35–40]. Additionally, G-protein mediated signaling involves more regulators such as GTPase activating proteins, phosducins and other proteins fine-tuning this pathway [41]. The genome of *T. reesei* comprises seven RGS domain containing proteins, of which four represent RGS proteins and three proteins are related to RGS-domain containing GprK-type GPCRs [42]. All RGS proteins of *T. reesei* contain a RGS box (130 amino acid motif; IPR016137) which is important for G-alpha binding [41].

Generally, the functions of RGS proteins in fungi range from pheromone response, growth and sporulation, pathogenicity [43, 44] and toxin production [45] to nematode trapping by *Athrobotrys* [46]. Due to their central functions in the physiology of fungi, they emerged also as important drug targets [47]. *T. reesei* RGS4 is related to *Aspergillus fumigatus* RgsC which is involved in vegetative growth and development, stress tolerance and virulence [48]. The *A. fumigatus rgsC* deletion mutant shows significantly decreased conidiophore formation and slower colony growth on plates but elevated spore germination on different carbon sources suggesting an involvement in the control of the cAMP/PKA pathway as well as a decreased tolerance to oxidative stress [48]. The down-regulation of gliotoxin (GT) genes and decreased GT production in *A. fumigatus* in mutants lacking *rgsC* might be due to the regulation of a global secondary metabolite regulator LaeA by RgsC [48].

In this study, we aimed to gain insight into the network of nutrient sensing and light response in *T. reesei*. Therefore, we investigated the role of RGS4, as a potential modulator of the activity of one or more of the three G-alpha subunits of *T. reesei*. We show here, that RGS4 impacts the physiology of *T. reesei* on multiple levels and that its major function occurs in light. RGS4 supports cellulase production, contributes to regulation of growth on several carbon sources and importantly it is required for proper gene regulation targeting iron homeostasis in light.

## Results

### *T. reesei* RGS4 is a typical member of the SNX/H group of RGS proteins

In *T. reesei* RGS4 (TrG0496W/TR_65607) is the homolog to *A. nidulans* RgsC and similar to other fungal proteins of this group (Additional file 1, Figure S1). The protein RGS4 contains two transmembrane regions (297–314 and 321–343 aa), a RGS domain (703–843 aa, E-value: 1.65e-19), a coiled coil (1108–1146 aa) and a PhoX homologous (PX) domain (1156–1269 aa, *E*-value 8.55e-25). This domain structure identifies RGS4 as a member of the subfamily of SNX/H RGS proteins [49]. If the Gs-alpha specificity is conserved in fungi, it is likely specific to the G-alpha s subunit GNA3 of *T. reesei* [37]. Hence, deletion of RGS4 may lead to enhanced or prolonged activation of GNA3. Checking available transcriptome data showed that *rgs4* is not significantly regulated in response to light, different carbon sources or during mating [24, 40, 50–52].

The regulation mechanism via phosphorylation is reflected in the amino acid sequence of RGS4 in that it comprises numerous protein kinase C (PKC) and casein kinase II (CKII) phosphorylation sites, both of which are associated also with light response processes [22, 53, 54]. The presence of four cAMP dependent protein kinase A (PKA) sites supports a potential connection to light signaling, since PKA is known as a priming kinase for casein kinase phosphorylation associated with light response [23]. RGS4 comprises three overlapping sites for PKA and CKII, which suggests a function of PKA as a priming kinase with RGS4, since PKA showed a light dependent function in cellulase regulation as well as generally in gene regulation also in *T. reesei* [55, 56]. However, this connection remains to be confirmed.

### RGS4 has its main function in light and is required for proper growth on glucose

**Fig. 1:**
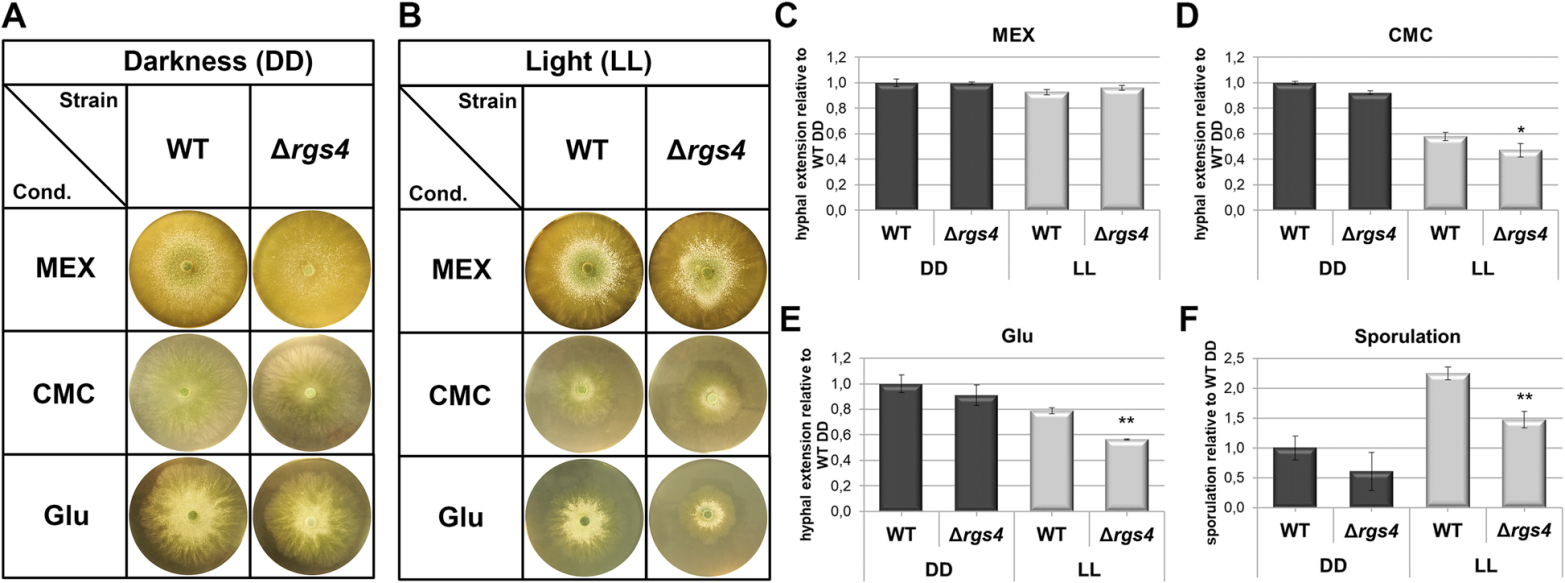
Influence of RGS4 on growth and asexual development. **A-E** Hyphal extension of wild-type (QM6a) and Δ*rgs4* on 3% malt extract (MEX) and Mandels-Adreotti minimal (MA) medium with 1% glucose and 1% carboxymethyl cellulose (CMC) as carbon source after 48 h in constant darkness (DD) and constant light (LL; 1700 lx). **F** Average sporulation measured after 48 h at 28 °C in DD and LL on 3% MEX. Measurements were taken from 3 biological replicates and statistical significance was calculated for the respective light condition (DD or LL) between WT and mutant using Student’s T-test. * = *p*-value < 0.05, ** = *p*-value < 0.01)

We deleted *rgs4* in the QM6a wild-type background, which resulted in viable deletion strains. G-protein signaling influences growth and the transmission of a cellulose related signal which is received via the class XIII GPCRs CSG1 and CSG2 and regulated by light in *T. reesei* [35]. Therefore, we analyzed hyphal apical extension rates of Δ*rgs4* on rich medium (3% malt extract, MEX) versus minimal medium (MA-medium) complemented with carboxymethyl cellulose (CMC) or glucose as the carbon source in constant light and constant darkness (Fig. 1). On malt extract we did not see any difference (Fig. 1A – C), whereas the deletion of *rgs4* led to a significantly decreased colony size on cellulose and glucose in light. In darkness, a small decrease (to 90%) in apical extension of Δ*rgs4* was detected*.* In light, colony sizes reached 70 to 80% of the wildtype on glucose or cellulose, respectively (Fig. 1D, E).

### RGS4 is involved in regulation of asexual development

It is well known that the cAMP and the heterotrimeric G-protein pathway play a crucial role in sporulation in fungi [57, 58]. In *T. reesei* QM6a sporulation is enhanced in light compared to dark grown cultures. We found that deletion of *rgs4* led to significantly decreased sporulation in light (Fig. 1F), whereas, in darkness, a negative trend was observed. We conclude that RGS4 is required for normal sporulation in *T. reesei*.

### RGS4 is required for proper stress response

**Fig. 2:**
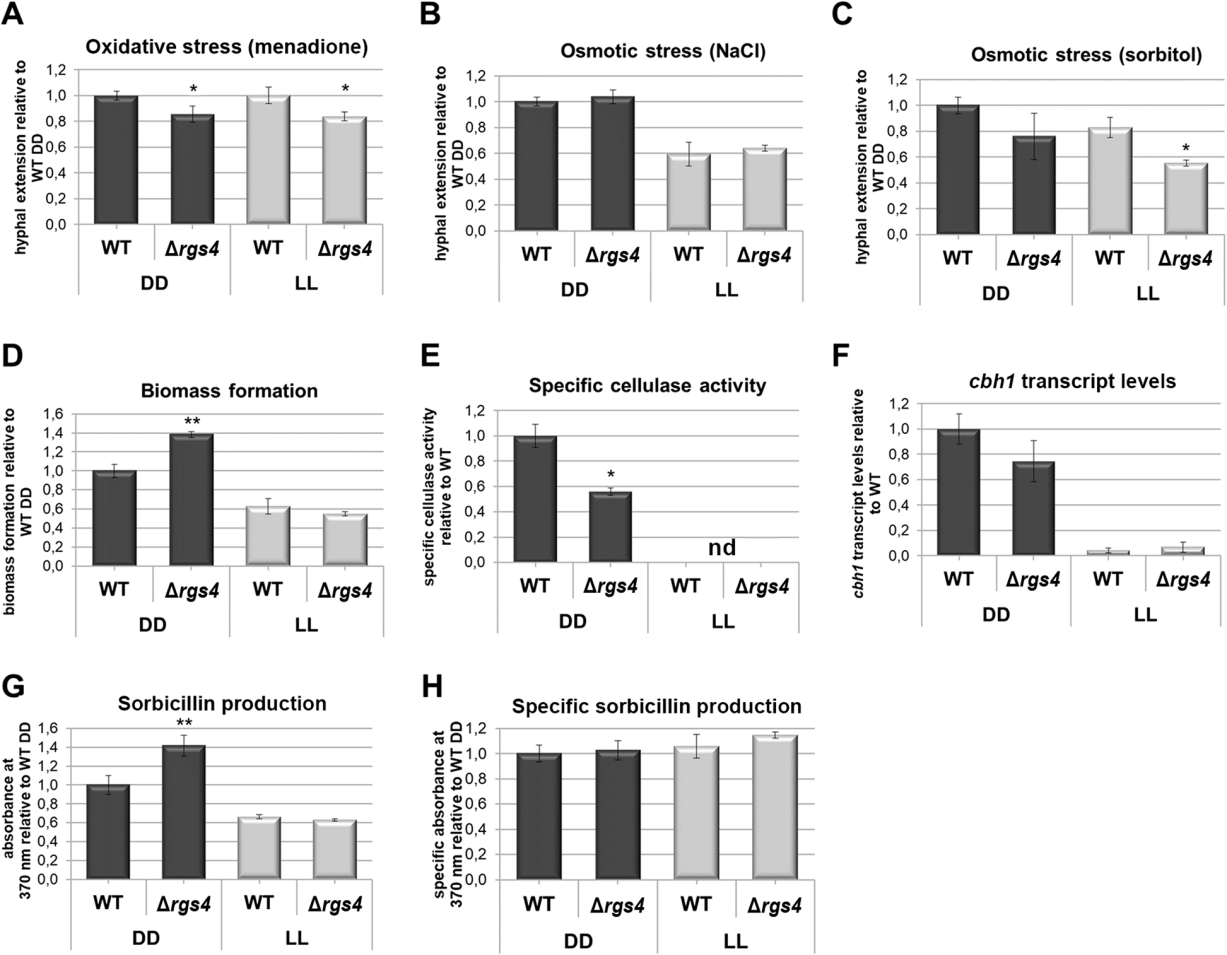
Relevance of RGS4 for stress response, growth, enzyme production and secondary metabolite biosynthesis. **A**-**C** Average hyphal extension after 96 h at 28 °C in constant darkness (DD) and light (LL) on MA-medium with 1% cellulose (CMC) as carbon source and supplemented with **A** 0.25 mM menadione or **B** 1 M NaCl or **C** 1 M sorbitol to test for reaction to oxidative or osmotic stress respectively. **D**-**H** Liquid cultivation at 28 °C after 96 h in constant light (LL) and darkness (DD). **D** Average biomass formation, **E** specific cellulase activity, **F ***cbh1* transcript levels (RT-qPCR) and **G** sorbicillin production represented as absorbances at 370 nm [59]. **H** Specific sorbicillin abundance in supernatant related to biomass formation upon growth on 1% cellulose. Measurements were taken from 3 biological replicates and statistical significance was calculated for the respective light condition (DD or LL) between WT and mutant using Student’s T-test.. * = *p*-value < 0.05)

For the RGS4 homologue in *A. fumigatus*, RgsC, hypersensitivity to oxidative stress on menadione, a natural organic compound that exerts its toxicity through the generation of reactive oxygen species (ROS), and reduced tolerance to the presence of H_2_O_2_ or paraquat was shown [48]. Therefore, we were interested in the role of RGS4 in oxidative stress response in *T. reesei* and found a significant decrease (*p* < 0.05) in resistance to menadione. In Δ*rgs4* the hyphal apical extension was significantly decreased in light and darkness compared to wild-type after 96 h on MA-CMC plates supplemented with 0.25 mM menadione (Fig. 2A). Since the control without menadione (Fig. 1D) did not show a significant growth defect under these conditions in darkness, RGS4 is concluded to contribute to resistance against oxidative stress in darkness. In light, the growth defect of Δ*rgs4* upon growth in the presence of menadione is in the range of the growth defect without oxidative stress (around 80% in both cases; Fig. 1D). Consequently, if there is a contribution of RGS4 to oxidative stress response in light, it is rather minor.

In *T. reesei* an involvement in sensitivity to osmotic stress by the G-protein pathway was shown previously [38]. To test the role of RGS4 we measured hyphal extension rates after 96 h on MA-CMC plates supplemented with 1 M NaCl or 1 M sorbitol. Interestingly the deletion of *rgs4* caused increased sensitivity to sorbitol but not to NaCl in light (Fig. 2B, C). Comparison with growth in the absence of osmotic stress on CMC (Fig. 1D) showed a more severe growth defect of Δ*rgs4* in the presence of 1 M sorbitol in both light and darkness. In case of osmotic stress applied by 1 M NaCl, the growth defect seen in the control (Fig. 1D) without stress is alleviated upon deletion of *rgs4*. Hence RGS4 is involved in the reaction of *T. reesei* to osmotic stress, particularly in the presence of NaCl (salt stress) in light.

### RGS4 impacts biomass formation and cellulase activity in constant darkness

Environmental sensing in microbes is essential for an optimal distribution of resources between growth (biomass formation), enzyme production and biosynthesis of secondary metabolites, among others. Therefore, we asked whether RGS4 contributes to one or more of these tasks. Upon growth in liquid media with cellulose in light, no difference in growth was observed, whereas in darkness biomass formation of Δ*rgs4* significantly increased by almost 40% (Fig. 2D). Specific cellulase activity was below the sensitivity limit for all samples in light, indicating that the deletion of *rgs4* does not alleviate the block of cellulase formation in light. For dark grown cultures, we found that RGS4 is required for high level cellulase formation (Fig. 2E). Accordingly, transcript abundance of the major cellobiohydrolase *cbh1/cel7a* showed a negative trend in darkness (*p*-value 0.108) (Fig. 2F).

*Trichoderma reesei* secretes sorbicillin derivates which are responsible for the characteristic yellow color of cultivation supernatants and plates [60, 61]. Since production of these pigments as well as regulation of the responsible SOR cluster is carbon source and light dependent [16], we tested whether RGS4 might be involved in this regulation. We found that deletion of *rgs4* increased the amount of yellow pigment in darkness, however, this increase rather can be explained by the increased biomass formation under these conditions (Fig. 2G, H). Our transcriptome analysis showed that all seven genes of the sorbicillin cluster [16, 36, 60], including the transcription factors *ypr1* (TrE0665C/TR_102499) and *ypr2* (TrE0663W/TR_102497), were up-regulated between 1.4- and 2.3-fold in Δ*rgs4* (see below). But this can only be considered a positive trend, because the threshold set for statistical significance was mostly not met (padj < 0.05). This result is hence in agreement with the lack of alteration of yellow pigment formation in Δ*rgs4.*

### RGS4 impacts gene regulation mainly in light

Phenotypic analyses revealed that RGS4 differentially affects physiology of *T. reesei* in light and darkness. Moreover, clear light dependent effects were shown for the influence of the heterotrimeric G-protein signaling pathway on regulation of plant cell wall degradation [13]. We were hence interested which role RGS4 plays in this mechanism connecting light response and reaction to available nutrients. Therefore, we cultivated Δ*rgs4* on minimal medium with cellulose as carbon source in constant light and constant darkness and assessed alterations in gene expression compared to the wild-type in both conditions.

**Fig. 3:**
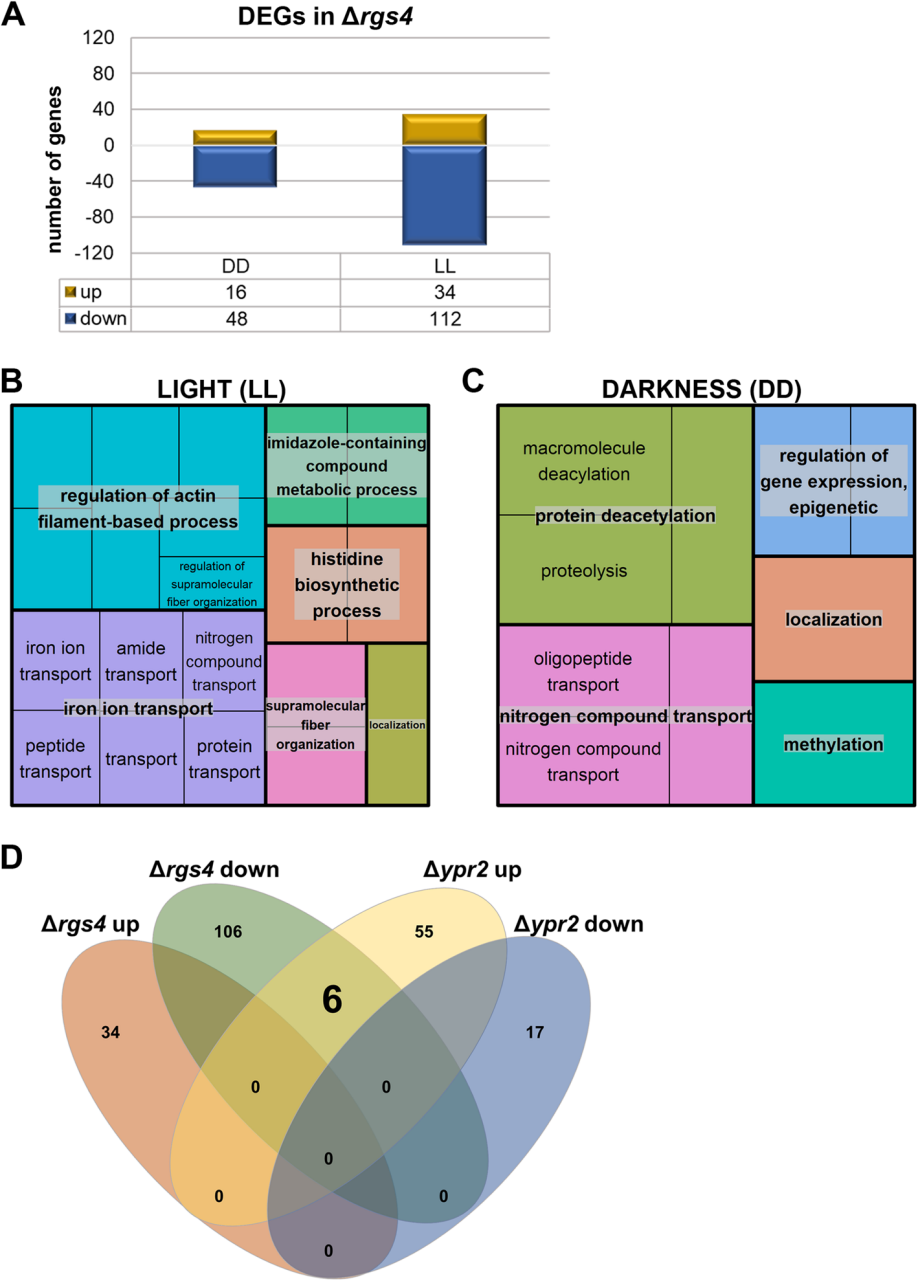
Gene regulation by RGS4 on cellulose in light and darkness. **A** Number of differentially expressed genes (DEGs) in Δ*rgs4. ***B**,** C** GO enrichment of DEGs visualized with REVIGO. **D** Number and overlap of DEGs in Δ*rgs4* and Δ*ypr2* in light

In Δ*rgs4* we found a total of 210 genes significantly differentially regulated (> 1.5-fold, padj < 0.05) of which 16 genes were up- and 48 down-regulated in darkness, and 34 up- and 112 down-regulated in light (Fig. 3A). Of those, three genes were regulated both in light and darkness by RGS4: a SANT domain transcriptional regulator TrB0388C/TR_4124 potentially involved in chromatin modification, which is significantly up-regulated on cellulose [35] and strongly down-regulated in light and a mutant lacking the sorbicillin transcription factor YPR2 [62] in darkness; a duf341 domain protein TrC1432W/TR_59368 with a comparable regulation pattern to TrB0388C/TR_4124 in light and on cellulose and an unknown unique secreted protein TrF0745W/TR_121883, which shows only minor light dependent regulation but an up-regulation on cellulose versus repressing/non inducing carbon sources [35].

For six of the differentially regulated genes, phosphorylation association with induction of plant cell wall degrading enzymes was detected [63]. They include genes encoding two predicted amino acid transporters (TrB0212C/TR_123718 up- and TrA0392C/TR_47175 down-regulated in light), a predicted plasma membrane H + ATPase (TrA2081W/TR_76238 up-regulated in light) and a ribosomal protein (TrB0953C/TR_47795 down-regulated in light). Additionally, four genes which are mutated in RutC30 (TrF004C/TR_79726, TrF0028C/TR_109211 and TrF0013C/TR_43418) including a muconate cycloisomerase gene (TrC0885C/TR_55887) showing regulation specific to cellulase inducing conditions [35] and two genes mutated in QM9123 (TrC0611W/TR_2439 and TrD0796W/TR_43191) were found among the genes down-regulated by RGS4 (Additional file 2).

In light, RGS4 is involved in regulation of transcript abundance of ribosomal protein genes. There were six ribosomal protein encoding genes down-regulated around twofold in the deletion mutant in light. Among which there were two 60S ribosomal protein genes *rla1* (TrB1847W/TR_123850) and *rla2* (TrD0208W/TR_123202), two potential small ribosomal protein genes *rps21* (TrB0594C/TR_78233) and *rps28* (TrC1311W/TR_106039), a potential mitochondrial ribosomal protein gene (TrC1283C/TR_121219) and a ribosomal protein gene TrB0953C/TR_47795. Additionally, we found a small nuclear ribonucleoprotein (snRNP) (TrA2076W/TR_43225) and a snRNA associated protein TrA1443W/TR_76073.

In constant darkness there are less genes differentially regulated in Δ*rgs4* as compared to in constant light but among those, categories involved in transport and localization stand out and can also be observed in the functional enrichment analysis (Fig. 3B, C). Out of 16 up-regulated genes in darkness, seven are transporters (also permeases or transferases) of which three are annotated as glutathione S-transferases (GST) [64]. Glutathione transferases belong to a protein family conserved across plants and animals, of detoxifying enzymes which are able to catalyze the conjugation of glutathione to form more soluble non-toxic compounds [65]. The number of GSTs in fungi correlates with the ability to degrade complex organic compounds and *T. reesei* was listed with the second highest number of GST genes present in the genome among fungi [66]. Among the down-regulated genes in darkness are two glycoside hydrolase genes (TrA0299W/TR_47268 (*bgl3i*) and TrF0168W/TR_65162) which is likely to contribute to the lower specific cellulase activity in Δ*rgs4.*

### RGS4 regulates a secondary metabolite cluster associated with siderophore production

**Fig. 4:**
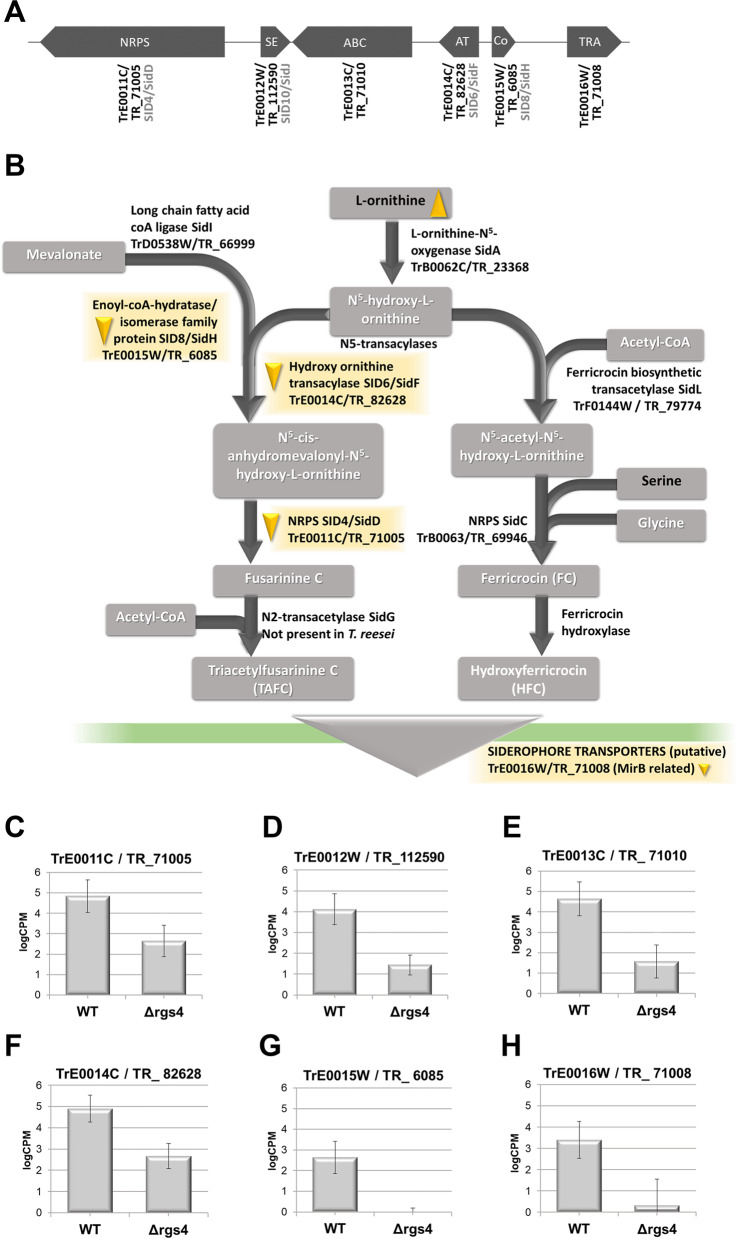
Regulation of a siderophore biosynthetic cluster by RGS4. **A** Schematic representation of the siderophore cluster in *T. reesei.* Model designations below the scheme taken from http://genome.jgi.doe.gov/Trire2/Trire2.home.html and if annotated, *T. reesei* protein names from Druzhinina et al. 2016 [64] and homologues names for *A. fumigatus* from https://fungidb.org/fungidb/app*.* NRPS (non-ribosomal peptide synthase), SE (siderophore esterase), ABC (ABC transporter), AT (acetyltransferase), Co (enoyl CoA hydratase), TRA (siderophore transporter). SID4/TrE0011C was former also known as TEX20. **B** Schematic representation of siderophore pathway and involved enzymes in *T. reesei.* Carbon sources analyzed by the BIOLOG Phenotype FF microarrays are given in black letters in grey boxes, compounds not analyzed are written in white. Downward pointing triangles indicate decreased growth in Δ*rgs4*, upwards pointing triangles indicate increased growth. Yellow triangles show growth differences in light (LL). Pathways and enzymes were taken from KEGG [64, 70, 71]. Model designations with the gene names taken from http://genome.jgi.doe.gov/Trire2/Trire2.home.html and if annotated, *T. reesei* protein names from Druzhinina et al. 2016 [64] and homologues names for *A. fumigatus* from https://fungidb.org/fungidb/app*. ***C**-**H** LogCPM normalized counts of siderophore cluster genes in wild-type and Δ*rgs4* in constant light (LL). Error bars show standard deviations. Statistical significance was calculated using Student’s T-test. * = *p*-value < 0.05

Among the down-regulated genes in light we found all six genes of a siderophore biosynthetic cluster (Fig. 4A, B): TrE0011C/TR_71005, TrE0012W/TR_112590, TrE0013C/TR_71010, TrE0014C/TR_82628, TrE0015W/TR_6085 and TrE0016W/TR_71008 (3.7 – 6.4-fold significantly down-regulated; Fig. 4C-H). TrE0015W is the homologue to *A. fumigatus* sidH, a mevalonyl CoA dehydratase, annotated in *T.reesei* as SID8, followed by a transacylase, SID6 (TrE0014C) and the NRPS siderophore synthase SID4 (TrE0011C) in the biosynthetic pathway. The genome of *T. reesei* does not comprise an N^2^-transacetylase gene, which would be responsible for acetylation of fusarinine C to triacetylfusarinine C (TAFC) (*A. fumigatus* sidG). However, for *A. fumigatus,* the production of fusarinine C seems to be sufficient as the major siderophore [67]. Additionally, also a siderophore transporter (TrE0016W) and an MDR type ABC transporter (TrE0013C) belong to this cluster and were found to be down-regulated as well as the iron transporter TrD0323C/TR_38812, not member of this cluster but indicative of an involvement of RGS4 in iron transport/synthesis in light, which is supported by the enriched functional category “iron transport” as well (Fig. 3B). In support of this hypothesis, also one of the multicopper oxidases of the reductive iron transport system, Fet3b (TrD0040C/TR_5119) was up-regulated in light. Regulation of an RGS protein in association with iron homeostasis has been shown for mammalian RGS19, which possesses a consensus iron-sulfur binding motif (CXXCXXC) [68, 69]. However, such a motif is not present in *T. reesei* RGS4.

On cellulose, already previously a light dependent regulation of this siderophore cluster upon growth on cellulose was found in the *ypr2* deletion mutant *in T. reesei* [62]. Therefore, we were interested if there is an overlap in regulatory targets between RGS4 and YPR2. YPR2 is a transcription factor located in the sorbicillin (SOR) cluster [60] and when deleted, the entire siderophore cluster was up-regulated in light, which contrasts with Δ*rgs4* where the siderophore cluster was down-regulated in light (Figs. 3D and 4C-H) [62]. Nevertheless, we did not detect mutual regulation of *ypr2* by RGS4 or vice versa. Interestingly, the six genes of the siderophore cluster were the only ones up-regulated in Δ*ypr2* and down-regulated in Δ*rgs4* in light (Fig. 3D). Consequently, the regulatory pathways involving YPR2 and RGS4 act in opposite directions concerning siderophore regulation.

To support the relevance of RGS4 for siderophore production we analyzed their presence in the supernatants of cellulose grown cultures. However, we saw that siderophore production upon growth on cellulose appears to be only slightly above the detection limit of the method and while we did observe a negative trend for Δ*rgs4* in light (data not shown), we consider gene regulation and growth patterns as more relevant evidence (see below).

### RGS4 impacts growth on diverse carbon sources

As our transcriptome analysis indicated that RGS4 is involved in regulation of metabolism, we asked whether this impact extends to regulation of growth. We therefore applied the BIOLOG FF Phenotype microarray system and tested growth on 95 carbon sources in constant light and constant darkness (Additional file 3). Measurements were taken from 72 to 144 h to cover peak biomass values for most of the carbon sources. Results were considered relevant if at least two consecutive measurements showed statistically significant differences to the wild-type (*p*-value < 0.05).

**Fig. 5:**
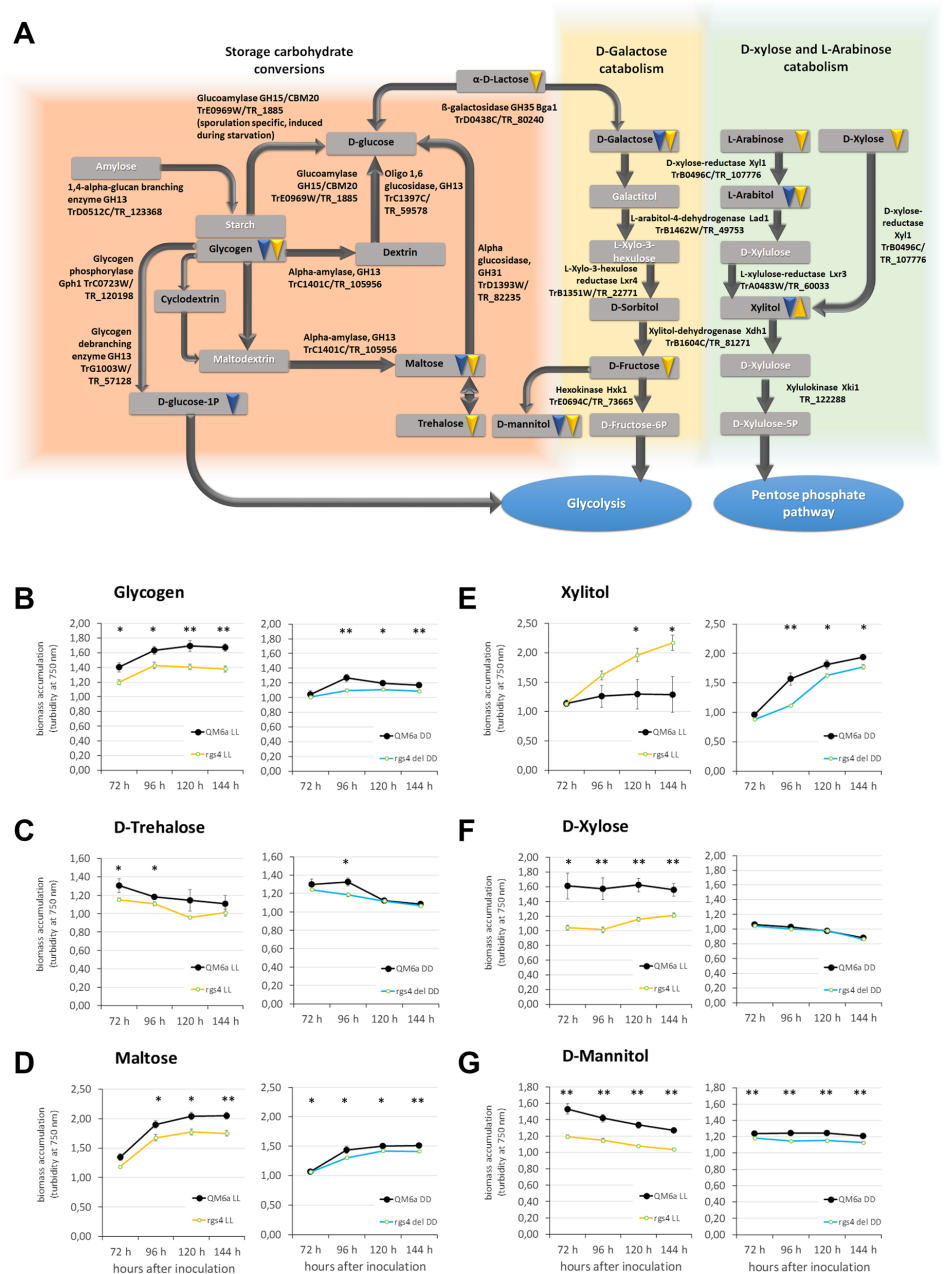
Biomass formation of Δ*rgs4* versus WT on carbon sources related to storage and sugar catabolism. **A** Schematic representation of carbohydrate conversion pathways and involved enzymes. Carbon sources analyzed by the BIOLOG Phenotype FF microarrays are given in black letters in grey boxes, compounds not analyzed are written in white. Downwards pointing triangles indicate decreased growth, upwards pointing triangles indicate increased growth. Blue triangles stand for growth in darkness (DD), while yellow triangles show growth differences in light (LL). Pathways and enzymes were taken from KEGG [71]. Gene model designations taken from http://genome.jgi.doe.gov/Trire2/Trire2.home.html and if annotated, *T. reesei* protein names from Druzhinina et al. 2016 [64] and homologues names for *A. fumigatus* from https://fungidb.org/fungidb/app*. ***B**-**D** Growth patterns of WT and Δ*rgs4* on storage related carbon sources i. e. **B** glycogen, **C** D-trehalose and **D** maltose in constant light (LL) or constant darkness (DD) as revealed by the BIOLOG system. **E–G** Growth patterns of WT and Δ*rgs4* on carbon sources representing intermediates of D-galactose, D-xylose or L-arabinose catabolism, i.e. **E** xylitol, **F** D-xylose or **G** D-mannitol. Error bars indicate standard deviation of three biological replicates. Asterisks show statistical significance of the difference between WT and Δ*rgs4* at a given time point (* = *p*-value < 0.05, ** = *p*-value < 0.01)

We found that growth on several storage carbohydrates is decreased in Δ*rgs4*. Growth defects in Δ*rgs4* were observed on dextrin, glycogen and trehalose, as is growth on the intermediate maltose, mostly in light upon lack of RGS4 (Fig. 5A-D). This finding suggests, that RGS4 promotes carbon storage degradation for increasing its biomass production. The decreased growth may hence reflect rerouting of these resources to other metabolic needs.

The observed growth patterns further suggest that RGS4 is involved in regulation of D-xylose, L-arabinose and D-galactose catabolism, as Δ*rgs4* grows more slowly on several intermediate carbohydrates of this pathway (Fig. 5A, E–G). In particular, growth on xylitol decreased in the dark, but increased in light (Fig. 5E), reflecting a light dependent regulation of the involved pathways by RGS4. Interestingly, this is not the case for D-xylose (Fig. 5F), which is converted to xylitol (Fig. 5A), as this shows the opposite effect in light (Fig. 5F). Consequently, we assume that due to the function of RGS4, xylitol conversion is promoted and upon deletion of *rgs4*, this intermediate is available for biomass production. In case of D-galactose, L-arabitol and D-mannitol (Fig. 5A), decreased growth was observed in both darkness and light, hinting at a more general effect of RGS4 targeting growth on these carbon sources.

Screening the transcriptome data for correlations of gene regulation with these growth patterns, we did not find regulations of the genes involved in the degradation pathways of these carbon sources. Consequently, we assume an impact of RGS4 is likely not at the transcriptional level but rather on a posttranscriptional level or that the targeted pathways are not operative or regulated upon growth on cellulose.

### RGS4 impacts growth on siderophore related carbon sources

**Fig. 6:**
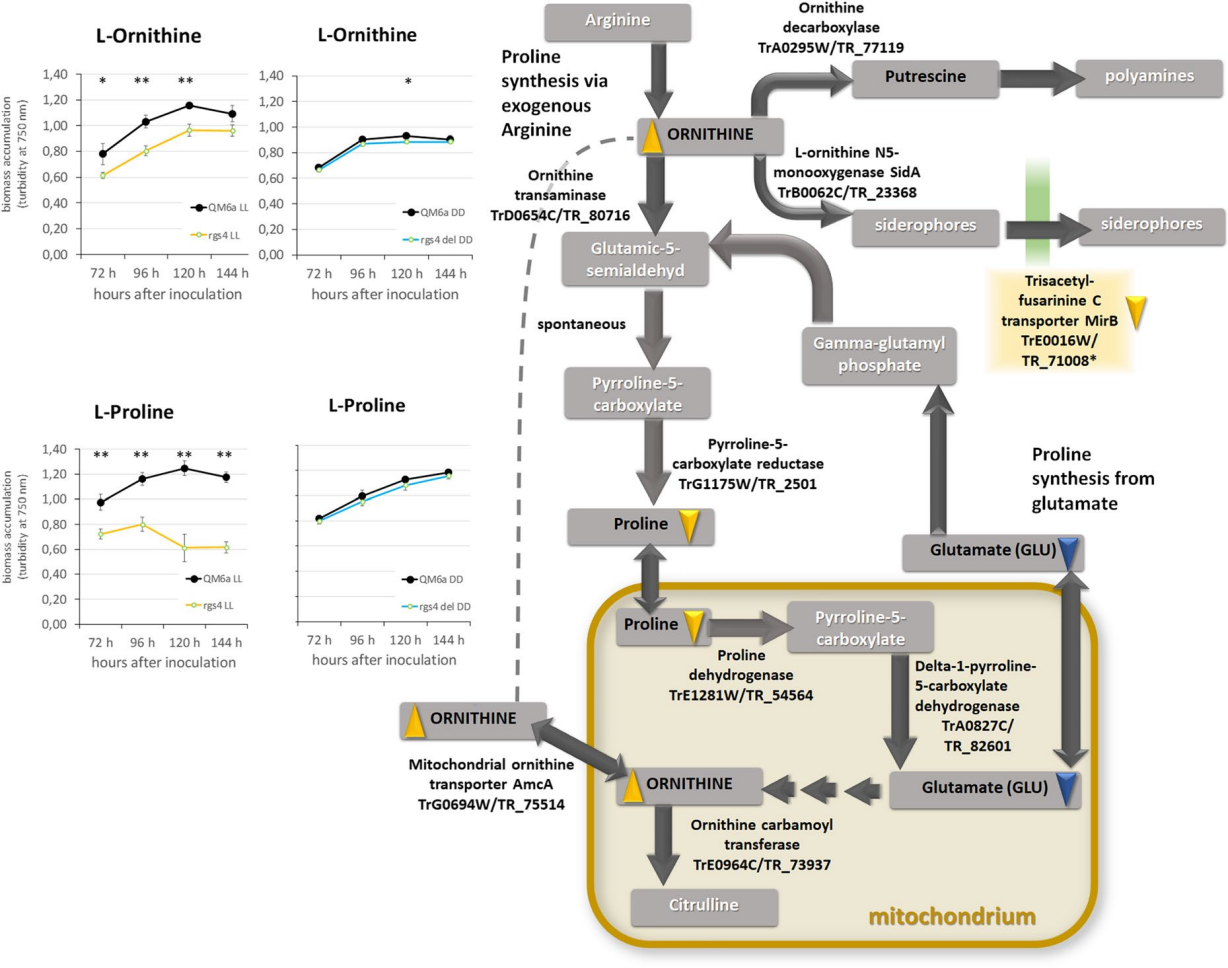
Biomass formation of Δ*rgs4* versus WT on carbon sources related to ornithine metabolism. **A**, **B** Growth patterns of WT and Δ*rgs4* on siderophore precursor (**A**) ornithine and amino acid (**B**) proline in constant light (LL) or constant darkness (DD) as revealed by the BIOLOG system. Error bars indicate standard deviation of three biological replicates. Asterisks show statistical significance of the difference between WT and Δ*rgs4* at a given time point (* = *p*-value < 0.05, ** = *p*-value < 0.01). **C** Schematic representation of conversion pathways and involved enzymes. Carbon sources analyzed by the BIOLOG Phenotype FF microarrays are given in black letters in grey boxes, compounds not analyzed are written in white. Downwards pointing triangles indicate decreased growth, upwards pointing triangles indicate increased growth; blue triangles stand for growth in darkness (DD), while yellow triangles show growth differences in light (LL). Pathways and enzymes are taken from KEGG [71–73]

The most interesting finding of the BIOLOG assay was the detection of carbon source utilization patterns supporting regulation of siderophore biosynthesis and indirectly iron homeostasis by RGS4 (Fig. 6). Importantly, growth on L-ornithine, the central precursor of siderophores, decreased in light, but not in darkness (Fig. 6A). Also, growth on L-proline decreased in light (Fig. 6B), although growth on glutamate only decreased in darkness. Since growth on putrescine, which is the intermediate in the metabolic pathway yielding polyamines, did not change in Δ*rgs4*, we assume that lack of *rgs4* decreases the consumption of proline, which may free resources for biomass production on ornithine. As an organic compound, the amino acid proline can be used as carbon and nitrogen source. In *A. fumigatus*, deletion of *rgsC*, the homologue of *rgs4*, resulted in restricted growth with proline as nitrogen source [48]. This is in agreement with our data, considering a role of proline as carbon source as well.

Additionally, decrease of transcript abundance and hence likely decrease of expression of the siderophore biosynthetic gene cluster and its operation in light also decreases conversion of ornithine for their production, again liberating resources for growth (Fig. 6C).

Inspection of the assay plates at the end of the experiment did not indicate significant differences in sporulation on one of the specific carbon sources tested.

## Discussion

**Fig. 7:**
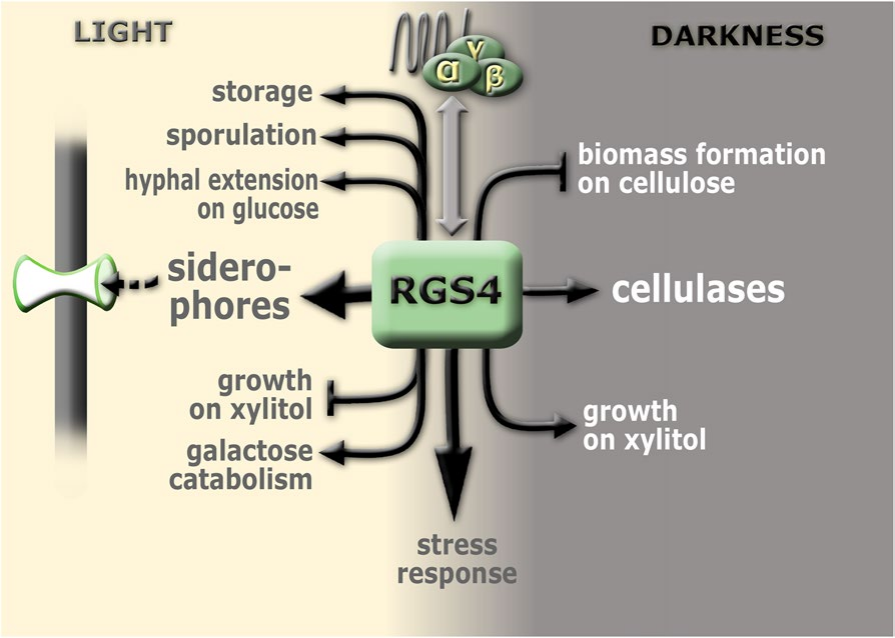
Schematic representation of the regulatory role of RGS4 in light and darkness. In light, *rgs4* is required for normal expression of the siderophore cluster producing fusarinine C and for expression of (iron) transporter genes. Furthermore, *rgs4* is positively involved in growth on storage- and galactose metabolism related carbon sources, sporulation and hyphal extension on glucose but negatively effects growth on xylitol as carbon source. In darkness on the other hand *rgs4* positively influences growth on xylitol and specific cellulase activity but decreases biomass formation on cellulose. In both, light and darkness, *rgs4* contributes to resistance against oxidative stress

It is crucial for fungi to sense and quickly adapt to their environment which relies on efficient signal transmission pathways. One of those pathways involves heterotrimeric G-protein signaling which is conserved in eukaryotes with its main components: the heterotrimeric G-proteins, G-protein coupled receptors (GPCRs) and regulators of G-protein signaling (RGS) [3, 74]. In *T. reesei*, roles of the G-protein α, β and γ subunits and a few GPCRs in the regulation of carbon or secondary metabolism in a light dependent manner was previously described [36–38, 40, 51]. RGSs on the other hand are still missing in this picture in *T. reesei* although they play an important role in the termination of signal from the Gα subunits. RGS proteins, just as G-proteins themselves, play important roles in the regulation of basic fungal processes such as vegetative growth, conidiation, secondary metabolite production and mating [41, 43]. In *A. fumigatus* the RGS proteins have been described in more details over the last years including *rgsC* which is involved in growth and development, tolerance to oxidative stress, gliotoxin production, expression of transporters and nutrient sensing [48]. To better understand the roles of RGS proteins specifically in light dependent regulation in *T. reesei* the current study provides insights into physiological changes and differential gene expression of RGS4 (Fig. 7)*.* One of the most interesting findings of this study is the difference of the regulatory targets of RGS4 in light versus darkness, which was not shown before and agrees with the light dependent role of the G-protein pathway shown previously [13, 37, 38].

In a previous study, a light independent regulation of growth by the Gα subunit 1 (*gna1*) on solid media with glucose as carbon source can be seen (slightly decreased hyphal extension of Δ*gna1* on glucose) [38]. Interestingly for Δ*rgs4* the effect is stronger in light and abolished in darkness indicating that RGS4 is light dependently required for the transmission of a glucose signal. In the same study [38] a light dependent involvement of GNA1 in the reaction to oxidative stress by menadione was shown. In constant light, deletion of *rgs4* caused a similar phenotype with decreased growth due to menadione, like the constitutive activation of GNA1, whereas in darkness we saw opposite effects: increased tolerance in both mutants of GNA1 and decreased tolerance in Δ*rgs4.* The deletion of an RGS protein should increase the signal strength of a Gα subunit because the intrinsic GTPase activity is not accelerated, however, in *T. reesei* there are four different free RGS proteins and it is not yet known whether their functions are redundant and how specific their interaction with the respective G-alpha subunits is. Considering the domain composition of RGS4, it would be predicted to act on the G-alpha s protein GNA3 rather than on GNA1 [49]. If this would be the case, strains lacking *rgs4* should have a phenotype resembling that of a constitutive activation of GNA3 (GNA3QL), which was reported earlier [37]. However, despite the confirmed function of RGS4 in cellulase regulation, the strong increase in transcript levels of the major cellulolytic enzyme encoding gene *cbh1* in light, which was earlier observed for GNA3QL [37] was not observed in Δ*rgs4.* This finding either suggests that the role of RGS protein in fungi is not entirely conserved or rather that investigation of G-protein function in the background of the high-cellulase random mutant QM9414 and its derivative TU-6 may be slightly different from that in the wild-type QM6a.

A light and carbon source dependent involvement of a GPCR, i.e. *gpr8,* in regulation of secondary metabolism was investigated previously, showing a decrease in transcript levels of SOR cluster genes and secondary metabolites produced in darkness on cellulose [36]. In part, the regulation patterns of GPR8 overlap with those of YPR2, a transcription factor located in the SOR cluster, but targeting a broad range of secondary metabolite biosynthesis genes [62]. Interestingly, in darkness two thirds of all of the genes differentially regulated in Δ*rgs4* are also differentially regulated in Δ*ypr2* but only six genes overlap in light, which all belong to the same siderophore cluster. This indicates a contribution of RGS4 in the initiation of a cascade that involves YPR2. The effect on siderophore regulation in light is opposite in both deletion mutants: RGS4 is required for siderophore gene cluster expression and YPR2 down regulates the cluster. Until now no direct correlation between RGS and iron transport has been shown in fungi, but in HELA cells the role of a RGS protein in the signaling cascade of iron chelation was shown [68].

Iron is essential for all eukaryotes and abundant on earth, but in an aerobic environment usually present in its oxidized form of ferric oxide hydrate complexes (Fe_2_O_3_ x nH_2_O) which has a low solubility of 10^–9^ to 10^–18^ M at neutral pH [67, 75]. Therefore, microbes had to develop different strategies for efficient iron uptake. One such a mechanism is siderophore-mediated Fe^3+^ uptake. Siderophores are low molecular mass iron chelators which help with the transport and storage of iron in the cell [76]. *A. fumigatus* acquires extracellular iron by a mechanism called reductive iron assimilation (RIA) [70]. During lack of extracellular and intracellular siderophores, *A. fumigatus* operates the RIA pathway, where ferric iron gets reduced to its ferrous form and is taken up by the FtrA/FetC complex [77, 78]. Defects in the RIA pathway cause an increase of siderophore production in *A. fumigatus* [79]. The genome of *T. reesei* comprises two Ftr1/Fet3 pairs, which are each located in vicinity to each other [62]. The respective gene pairs encoding FET3a/FTR1a and FET3b/FTR1b are co-regulated and *fet3a/ftr1a* show increased transcript levels in light on cellulose, while *fet3b/ftr1b* transcript abundance is decreased in light [62]. These data indicate that the two distinct gene pairs involved in the reductive iron uptake system in *T. reesei* confer light dependent specificity of this process, which likely also influence siderophore regulation. In our study, only *fet3b* was up-regulated in light in Δ*rgs4.* We conclude that an influence of RGS4 on siderophore production and precursor metabolism in light but not in darkness is in agreement with the hypothesis of a light dependent relevance of iron as a nutrient, but also as a signal.

Phosphorylation is considered the currency of signal transduction cascades [80]. In recent years it was confirmed that fungi react to the presence of plant cell wall carbohydrates with phosphorylation of diverse proteins, including those within signal transduction cascades [81, 82]. This response happens within minutes of recognition of altered environmental conditions and is both transient and dependent on the sensed carbon source [81, 82]. Although RGS4 does not regulate protein kinases at the transcriptional level, we found several genes encoding proteins specifically phosphorylated upon detection of residues associated with plant cell wall degradation [63] among the targets of RGS4.

As generally with phosphorylation [83], this posttranslational modification of transporters may impact activity, stability or conformation/sensitivity in dependence of the substrate to be transported. Interestingly, the *S. cerevisiae* homologue of one of the predicted amino acid transporters (TrA0390C/

TR_47175), Avt3p, is phosphorylated by the kinase Atg1p [84], hence supporting a conserved relevance of this modification. Considering that for only around 8% of predicted proteins of *T. reesei* phosphorylation (of one or more peptides) was detected [63], the finding of six genes with plant cell wall degradation associated phosphorylation among the targets of RGS4 only in light (3 up-regulated, 3 down-regulated) is remarkable.

With functions in stress response and regulation of the metabolism of storage carbohydrates, RGS4 modulates physiologically crucial mechanisms intimately associated with survival. Moreover, in both cases a role in reaction to changing or deteriorating environmental conditions is implicated by this function and as with other functions of RGS4, it is connected to a light dependent relevance. The decreased growth upon degradation of extracellular glycogen, dextrin or trehalose hints to a lower expression or secretion of the respective enzymes and a function of RGS4 balancing growth with storage of carbohydrates in response to the environment.

## Materials and methods

### Strains and cultivation conditions

For the genotype of all strains used in this study see Table 1. The wild-type strain referred to in this study is *T. reesei* QM6a [85] which was used as a parental strain to construct the recombinant strain QM6aΔ*rgs4*.

**Table 1:** Strains used in this study

Strain	Code	Characteristics	Source
QM6a	WT	Wild-type	[85]
FF1	FF1	Female fertile derivative of QM6a (MAT1-1)	[86]
FF2	FF2	Female fertile derivative of QM6a (MAT1-2)	[86]
QM6aΔ*rgs4*	Δ*rgs4*	Δ*rgs4*::hph^+^ in QM6a background	This study
FF2rgs4_P5, FF2rgs4_P7, FF2rgs4_P11	FF2Δ*rgs4*	backcrossed Δ*rgs4* with FF1, carrying the deletion	This study
FF2rgs4_DR_3, FF2rgs4_DR_4	FF2*rgs4*DR	backcrossed Δ*rgs4* with FF1, not carrying the deletion	This study

Liquid cultivation was performed in Mandels Adreotti minimal medium (MA medium; [87]) containing 1% (w/v) microcrystalline cellulose (Alfa Aesar, Karlsruhe, Germany) and 0.1% (w/v) peptone to induce germination in constant dark and constant light (1700 lx) for 96 h at 200 rpm and 28° C. Strains for cultivation (QM6a and Δ*rgs4*) were revived from glycerol stocks and then grown on 3% (w/v) malt extract agar (MEX) for 14 days in constant darkness which prevents interference of circadian rhythmicity with the analyses. 10^9^ conidia/L were used for the inoculation of 50 mL MA medium in shake flasks in triplicates. For harvest in darkness a very low red safety light (darkroom lamp, Philips PF712E, red, 15W) was used.

Phenotypic plate assays were analyzed after 48 h at 28° C under constant light (1700 lx) and constant darkness. Sporulation was measured in triplicates at 600 nm, which correlates with microscopic spore counts. After excision of an agar piece of defined size (2 × 1.77 cm^2^) from malt extract plates (3% w/v) spores were collected in 4 mL spore solution (0.8% w/v NaCl and 0.05% w/v Tween 80 in purified water) and photometrically analyzed at 600 nm against a standard curve of pre-counted spores.

Hyphal extension assays were analyzed upon growth on MA medium supplemented with either 1% w/v carboxymethyl cellulose (CMC) or 1% w/v glucose (Glc). For growth under stress, MA-CMC was supplemented with either 1 M sorbitol or 1 M NaCl for osmotic stress or 0.25 mM menadione (Sigma-Aldrich, St. Louis, Missouri, USA) for oxidative stress and measured after 96 h.

### Construction of Δ*rgs4*

The deletion mutant Δ*rgs4* was created by recombinant cloning using a hygromycin phosphotransferase (*hph*) marker cassette with 1 kilobases (kb) flanking regions produced by yeast recombination as described previously [88] and protoplast transformation was performed with selection plates supplemented with 50 µg/mL hygromycin B as selection reagent (Roth, Karlsruhe, Germany) [89]. Successful deletion was confirmed by PCR. For primer sequences see Table 2. Copy number determination by qPCR as described previously [52] indicated two copies of the *rgs4* deletion cassette. Consequently, we aimed to confirm that the observed effects are due to the deletion of RGS4 rather than random effects of transformation. We performed crosses of Δ*rgs4* with female fertile FF1 to obtain progeny carrying the deletion. Analysis of these strains lacking *rgs4* as well as progeny from this crossing in which the deletion had been restored, confirmed that the characteristic growth defect of Δ*rgs4* on glucose segregated with the deletion (Additional file 1, Figure S2) hence confirming the validity of the strain used for analyses.

**Table 2:** Oligonucleotides used in this study

Name	Sequence 5'—3'	Purpose
Rgs4_65607_5F	GTAACGCCAGGGTTTTCCCAGTCACGACGCCTGTTCAGAGCCTTATTCC	forward primer for 5' flank
Rgs4_65607_5R	ATCCACTTAACGTTACTGAAATCTCCAACGTACCGAGTACAAAACGTCG	reverse primer for 5' flank
Rgs4_65607_3F	CTCCTTCAATATCATCTTCTGTCTCCGACGAACCTGGTGTGATTTGAAGG	forward primer for 3' flank
Rgs4_65607_3R	GCGGATAACAATTTCACACAGGAAACAGCGGCATCCGTCCATAGTGAG	reverse primer for 3' flank
Rgs4_65607_qF	CGTGATACAGGAGAGCGATA	Internal primer
Rgs4_65607_qR	TTGGTGCAGTTCGTGAAAC	Internal primer
EF1-728F	CATCGAGAAGTTCGAGAAGG	Internal primer
TEF1 rev	GCCATCCTTGGAGATACCAGC	Internal primer
SAR RTF1	TGGATCGTCAACTGGTTCTACGA	RT qPCR
SAR RTR1	GCATGTGTAGCAACGTGGTCTTT	RT qPCR
RTcbh1F	ACCGTTGTCACCCAGTTCG	RT qPCR
RTcbh1R	ATCGTTGAGCTCGTTGCCAG	RT qPCR

### Isolation and manipulation of nucleic acids

DNA for screening of mutants was extracted using a rapid mini preparation method for fungal DNA [90]. For the isolation of total RNA, the mycelium from liquid cultivation was filtered through miracloth and frozen in liquid nitrogen prior to extraction with the RNeasy Plant mini kit (Qiagen, Heidelberg, Germany). Quality control of total RNA and RT-qPCR for investigation of *cbh1* transcript levels was performed as described earlier [91, 92]. *Sar1* was used as reference gene. Oligonucleotide sequences of all primers used in this study are listed in Table 2.

### Biomass determination and specific cellulase activity

Biomass was determined as described earlier [93]. Briefly, frozen mycelia from liquid cultivation were ground in liquid nitrogen, incubated in 0.1 M NaOH and sonicated to break up cells. The liberated protein content was then measured using the Bradford method as a means reflecting biomass content.

For the analysis of cellulases in the cultivation supernatant, after centrifugation to remove residual cellulose, the CMC-cellulose kit (S-ACMC-L Megazyme) was used to measure endo-1,4-ß-D-glucanases. For the specific cellulase activity, the cellulase activity was normalized to the biomass produced.

### Sorbicillin analysis at 370 nm

Absorbance at 370 nm reflects sorbicillin content [59] and was hence applied to quantitatively assess the amount of yellow pigment, indicative for sorbicillin and its derivatives in liquid media. Supernatants of liquid cultivation were centrifuged to remove residual cellulose and absorbance at 370 nm indicative for sorbicillin measured from biological triplicates.

### BIOLOG phenotype microplate assay

Growth on different carbon sources were analyzed using BIOLOG FF Microplate assay (Biolog Inc., Hayward, CA) as described previously [94]. Inoculated microplates were incubated at 28 °C in constant dark or constant light (1700 lx) for up to 144 h and absorbances measured at 750 nm reflecting biomass accumulation in 24 h intervals starting at 72 h. Analyses were repeated in triplicates for each strain. Statistical significance of growth differences was analyzed by the T-test (*p*-value threshold ≤ 0.05) as implemented in Excel 2016 (Microsoft, Redmond, USA).

### Transcriptome analysis and bioinformatics

We submitted total RNA in biological triplicates for each strain and condition. Library preparation, including ribo-depletion for the removal of rRNA and sequencing was conducted at the Next Generation Sequencing Facility (Vienna Biocenter Core Facilities GmbH, Austria) on a NovaSeq 6000 in paired-end (PE) and 150 bp mode, which resulted in an average of 29 million reads per sample. Quality filtering (Q30) and adapter trimming was done using bbduk version 38.18 [95]. For mapping, we used the most recent *T. reesei* QM6a reference genome [8] using HISAT2 version 2.2.1 [96], with an average overall alignment of 99.0% on average. For further data processing we used samtools version 1.10 [97] and examined the quality of mapping with QualiMap version 2.2.2 before applying featureCounts version 2.0.1 [98]. For differential gene expression (DEG) analysis in R version 4.0.3 [99], DESeq2 version 1.3.1 [100] was used with a threshold for significantly differentially regulated genes of log2fold change |> 0.58| and p-adj < 0.05. Resulting DEGs were further filtered with the LFCshrink function (type: apeglm) [101]. The gene annotations were done using available annotations for *T. reesei*, *T. virens* and *T. atroviride* [42] and *T. reesei* [64]. For count normalization the DESeq2 variance stabilizing transformation (VST) function was applied. Functional enrichment of a set of DEGs was performed using the Fisher’s exact test using R package topGO version 2.42.0 [102] visualized with REVIGO [103].

### Statistics

Statistical significance for phenotypic analysis was calculated in R using Student’s T-test (compare means, ggpubr version 0.4.0) ** = *p*-value < 0.01, * = *p*-value < 0.05.

Phylogenetic analysis was performed using clustalX [104] for the alignment and MEGA11 for minimum evolution analysis [105, 106].

## Supplementary Information

**Additional file 1.**

**Additional file 2.**

**Additional file 3.**

## Data Availability

The datasets generated and analyzed during the current study are included in this article and its additional files and under GenBank accession number GSE216955 (https://www.ncbi.nlm.nih.gov/geo/query/acc.cgi?acc=GSE216955).
