# Supplementary material for: RGS4 impacts carbohydrate and siderophore metabolism in Trichoderma reesei
Source: BMC Genomics. 2023 Jul 3;24:372. doi: 10.1186/s12864-023-09467-2 (PMC10316542; doi:10.1186/s12864-023-09467-2)
Supplement: Supplementary file 1 — Additional file 1. [file 12864_2023_9467_MOESM1_ESM.docx]

**RGS4 impacts carbohydrate and siderophore metabolism in *Trichoderma reesei***

Miriam Schalamun^1^, Eva Maria Molin and Monika Schmoll^1,2^*

^1^ AIT Austrian Institute of Technology GmbH, Center for Health and Bioresources, Bioresources Unit, Konrad Lorenz Strasse 24, 3430 Tulln, Austria

^2^ University of Vienna, Department of Microbiology and Ecosystem Science, Division of Terrestrial Ecosystem Research, Djerassiplatz 1, 1030 Vienna, Austria

**Supplementary material**


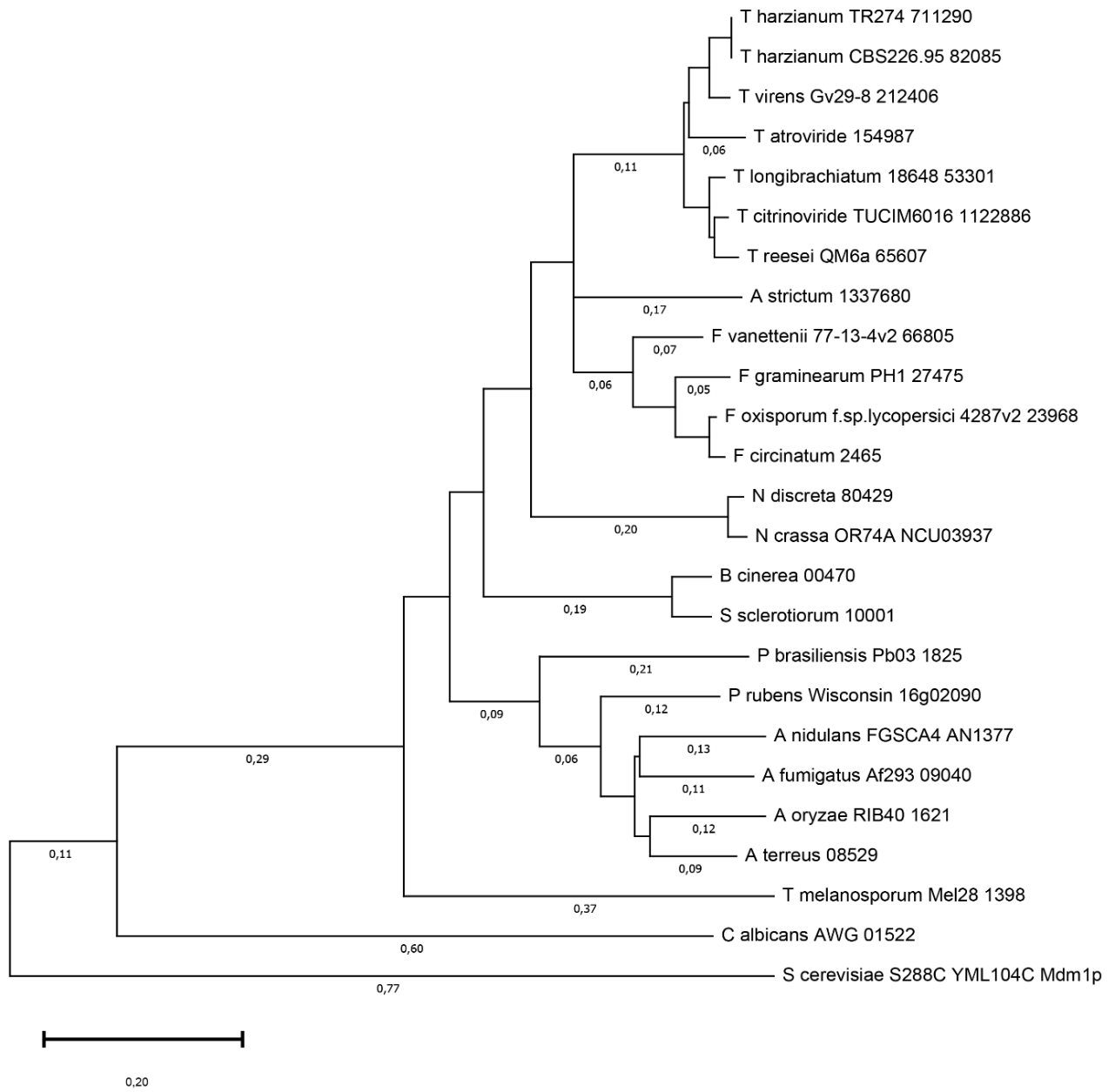


**Figure S1.** Phylogenetic analysis of RGS4 (TrG0496W) in fungi. Sequences were retrieved from fungiDB (https://fungidb.org/fungidb/app) and JGI (https://mycocosm.jgi.doe.gov/mycocosm/home). Species names, strain and protein IDs are provided. Sequence alignment was performed using ClustalX and phylogenetic analysis with MEGA11. Sequences used are: *Trichoderma harzianum, Trichoderma virens, Trichoderma atroviride, Trichoderma longibrachiatum, Trichoderma citrinoviride, Trichoderma reesei, Acremonium strictum, Fusarium vanettenii, Fusraium graminearum, Fusarium oxisporum, Fusarium circinatum, Neurospora discreta, Neurospora crassa, Botrytis cinerea, Sclerotinia sclerotiorum, Paracoccidioides brasiliensis, Penicillium rubens, Aspergillus nidulans, Aspergillus fumigatus, Aspergillus oryzae, Aspergillus terreus, Tuber melanosporum, Candida albicans, Saccharomyces cerevisiae*.


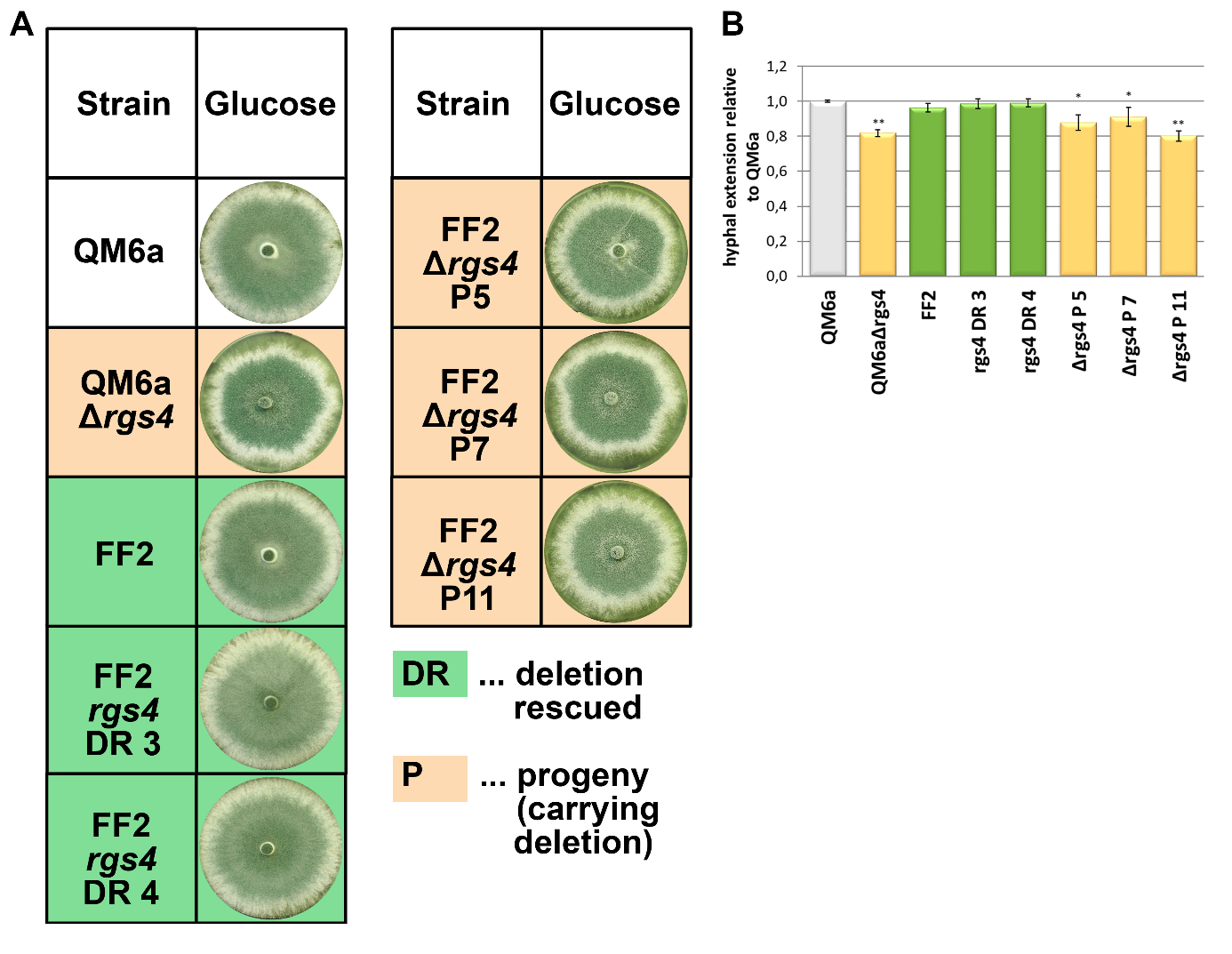


**Fig S2.** Hyphal extension of wild types QM6a and FF2, QM6aΔ*rgs4* and progeny of Δrgs4, carrying the deletion (rgs4_P5, rgs5_P7, rgs4_P11) and progeny not carrying the deletion (rgs4_DR3 and rgs4_DR4). Grown on MA-medium with 1% glucose under constant light. (**A**) after 96h, (**B**) after 48h.
